# Neuropathological lesions in intravenous BCG-stimulated K18-hACE2 mice challenged with SARS-CoV-2

**DOI:** 10.1186/s13567-024-01325-7

**Published:** 2024-05-31

**Authors:** Lidia Sánchez-Morales, Néstor Porras, Teresa García-Seco, Marta Pérez-Sancho, Fátima Cruz, Blanca Chinchilla, Sandra Barroso-Arévalo, Marta Diaz-Frutos, Aránzazu Buendía, Inmaculada Moreno, Víctor Briones, María de los Ángeles Risalde, José de la Fuente, Ramón Juste, Joseba Garrido, Ana Balseiro, Christian Gortázar, Antonio Rodríguez-Bertos, Mercedes Domínguez, Lucas Domínguez

**Affiliations:** 1 VISAVET Health Surveillance Centre, Complutense University of Madrid, 28040 Madrid, Spain; 2 Department of Animal Health, Faculty of Veterinary Medicine, Complutense University of Madrid, 28040 Madrid, Spain; 3 Department of Animal Production, Faculty of Veterinary Medicine, Complutense University of Madrid, 28040 Madrid, Spain; 4 Unidad de Inmunología Microbiana, Centro Nacional de Microbiología, Instituto de Salud Carlos III, Carretera Pozuelo-Majadahonda km 2, Majadahonda, 28220 Madrid, Spain; 5 Departamento de Anatomía y Anatomía Patológica Comparadas y Toxicología, Grupo de Investigación en Sanidad Animal y Zoonosis (GISAZ), UIC Zoonosis y Enfermedades Emergentes (ENZOEM), Universidad de Córdoba, Córdoba, Spain; 6 SaBio Instituto de Investigación en Recursos Cinegéticos, Ciudad Real, Spain; 7 Department of Veterinary Pathobiology, Center for Veterinary Health Sciences, Oklahoma State University, Stillwater, OK USA; 8 Animal Health Department, NEIKER-Basque Institute for Agricultural Research and Development, Basque Research and Technology Alliance (BRTA), 48160 Derio, Bizkaia Spain; 9 Departamento de Sanidad Animal, Facultad de Veterinaria, Universidad de León, 24071 León, Spain; 10 Department of Internal Medicine and Animal Surgery, Faculty of Veterinary Medicine, Complutense University of Madrid, 28040 Madrid, Spain; 11 Real Academia de Doctores de España, C. de San Bernardo, 49, 28015 Madrid, Spain

**Keywords:** BCG stimulation, SARS-CoV-2, K18-hACE2, neuroinvasion

## Abstract

In the wake of the COVID-19 pandemic caused by SARS-CoV-2, questions emerged about the potential effects of Bacillus Calmette-Guérin (BCG) vaccine on the immune response to SARS-CoV-2 infection, including the neurodegenerative diseases it may contribute to. To explore this, an experimental study was carried out in BCG-stimulated and non-stimulated k18-hACE2 mice challenged with SARS-CoV-2. Viral loads in tissues determined by RT-qPCR, histopathology in brain and lungs, immunohistochemical study in brain (IHC) as well as mortality rates, clinical signs and plasma inflammatory and coagulation biomarkers were assessed. Our results showed BCG-SARS-CoV-2 challenged mice presented higher viral loads in the brain and an increased frequency of neuroinvasion, with the greatest differences observed between groups at 3–4 days post-infection (dpi). Histopathological examination showed a higher severity of brain lesions in BCG-SARS-CoV-2 challenged mice, mainly consisting of neuroinflammation, increased glial cell population and neuronal degeneration, from 5 dpi onwards. This group also presented higher interstitial pneumonia and vascular thrombosis in lungs (3–4 dpi), BCG-SARS-CoV-2 mice showed higher values for TNF-α and D-dimer values, while iNOS values were higher in SARS-CoV-2 mice at 3–4 dpi. Results presented in this study indicate that BCG stimulation could have intensified the inflammatory and neurodegenerative lesions promoting virus neuroinvasion and dissemination in this experimental model. Although k18-hACE2 mice show higher hACE2 expression and neurodissemination, this study suggests that, although the benefits of BCG on enhancing heterologous protection against pathogens and tumour cells have been broadly demonstrated, potential adverse outcomes due to the non-specific effects of BCG should be considered.

**Supplementary Information:**

The online version contains supplementary material available at 10.1186/s13567-024-01325-7.

## Introduction

Severe acute respiratory syndrome coronavirus 2 (SARS-CoV-2) is the causative agent for the global pandemic of coronavirus disease 2019 (COVID-19), which resulted in 759 million confirmed cases and 6.8 million deaths reported as of 2023 [1]. COVID-19 is a multi-organ disease, that presents a diverse array of symptoms. It is important to note that a substantial 36.4% of individuals who succumbed to COVID-19 experienced neurological manifestations [2], including intracranial haemorrhages, parkinsonism, sleep disorders, and symptoms akin to Alzheimer’s disease, such as cognitive deficits, seizures, delirium, and behavioral alterations [3]. Moreover, 30–60% of patients continue to experience neurological symptoms 6 months after their initial infection [4]. Additionally, individuals with pre-existing conditions such as Alzheimer’s disease or autism are at higher risk of contracting COVID-19 due to potential disruptions with the blood–brain barrier, which can also exacerbate their underlying conditions, potentially leading to increased mortality rates [5].

The mechanism of SARS-CoV-2 infection involves the binding of the virus to the angiotensin-converting enzyme (ACE2) receptor present in human cells [6]. Although ACE2 receptors are distributed across various tissues, immunohistochemistry (IHC) studies revealed marked ACE2 staining in type I and II lung alveolar epithelial cells [7]. Notably, investigations of human brain tissue have identified the presence of hACE2 (human angiotensin-converting enzyme 2) receptors in vascular endothelial and smooth muscle cells, both in the peripheral and central nervous system [8, 9]. This finding offers one of the potential explanations for how the virus enters the brain and leads to neurological clinical symptoms and lesions [10].

The severity of COVID-19 clinical signs varies among individuals and is influenced by several factors, including age [11], sex [12], comorbidities with other diseases [13], and even blood type [14]. These variables can either exacerbate or mitigate the severity of the disease and its clinical manifestations. Another intriguing factor is the impact of prior immunization with immunomodulatory agents like the Bacillus Calmette–Guérin (BCG) vaccine, which has been suggested to play a role on the SARS-CoV-2 infection [15]. This is the only approved anti-tuberculosis vaccine which was originally developed to combat tuberculosis in the twentieth century and has been already administered worldwide in more than 4 billion doses [16]. Besides its primary function, BCG exhibits immunomodulatory properties and offers protection against diseases like leprosy, non-tuberculous mycobacterial lymphadenitis, and Buruli ulcer [17]. It may also be associated with a lower risk on leukemia [18] and lung cancer [19], or a therapeutic effect bladder cancer [20]. Moreover, BCG induces trained immunity and non-specific protection against various infections, including respiratory tract infections and neonatal sepsis which protection can persist up to 1 year and involves increased production of proinflammatory cytokines [21].

The COVID-19 pandemic raised questions about whether BCG vaccination could enhance the immune response against SARS-CoV-2 [15, 22]. To explore this, population-based surveys studies were conducted [23, 24], suggesting that BCG vaccination may reduce the severity of COVID-19. However, there is some controversy, as other epidemiological studies identified BCG vaccination as a potential risk factor, increasing the likelihood of COVID-19 diagnosis, positive PCR tests, or hospitalization [25]. Similarly, certain studies have reported no decrease in SARS-CoV-2 infections [26]. In this way, the impact of BCG vaccination on COVID-19 remains uncertain and requires further investigation [27].

To address this uncertainty, the K18-hACE2 mouse model has emerged as a primary tool. These mice are transgenic and express the human hACE2 gene under the control of the cytokeratin-18 (k18) promoter [28]. This model has been frequently employed in initial investigations concerning the pathogenesis of SARS and SARS-CoV-2, as well as in the development of vaccines against these diseases [29]. However, it is important to consider the higher expression of ACE2 receptors and viral neurodissemination observed in this model in comparison to humans [30]. Recent research has explored brain lesions in K18-hACE2 transgenic mice resulting from SARS-CoV-2 infection and brain invasion, examining viral replication [31] and the chronological progression of histological lesions [32]. Additionally, many studies have explored the distribution of SARS-CoV-2 antigen and the impact on the olfactory bulb, as well as presence of anosmia in k18-hACE2, a common sign in human disease [33]. The results regarding BCG stimulation in this experimental model have been diverse, with some studies suggesting beneficial effects [30, 34] while others reported no discernible protection against SARS-CoV-2 [35, 36]. Other experimental models such as golden Syrian hamster have been also utilized, showing positive results for BCG vaccination in reducing the severity of SARS-CoV-2 infection [37]. A gap remains in the literature, as studies that integrate BCG administration prior to SARS-CoV-2 infection along with comprehensive histopathological study of brain lesions are lacking. This highlights a significant research void to clarify the potential association between BCG stimulation and neurological diseases together with SARS-CoV-2 infection, since BCG has already been related to some therapies against neurological illnesses [38].

The present study was conducted to offer a comparative analysis of the disease’s progression and central nervous system lesions as well as the effects BCG may have in both.

To our knowledge, this investigation contributes to the field with a comprehensive brain histopathological and IHC evaluation of BCG-stimulated K18-hACE2 mice post-SARS-CoV-2 challenge, providing new insights in this research area.

## Materials and methods

### Animals

Animal care and procedures were performed by following the guidelines of good experimental practices according to Directive 2010/63/EU of the European Parliament and of the Council of 22 September 2010 on the protection of animals used for scientific purposes [amended by the Regulation (EU) 2019/1010)] and Spanish laws (RD 53/2013). The protocol was approved by the Community of Madrid Ethics Committee (reference PROEX 180.2/22). K18-hAC2 male and female mice (*n* = 43) aged 4–6 weeks were obtained from Charles River Laboratories (Wilmington, Massachusetts, USA). The cages were equipped with Altromin-LASQCdiet® Rod 14-H (Altromin Lage, Germany) for feeding. Both food and water were available ad libitum. Wheeled houses for environmental enrichment were also included (Ref: K3327 + K3250, Sodispan Research, Madrid, Spain). During the initial 10 day-period, they were given time to acclimate to their new cages and socialize with their partners. After that period, the animals were marked with an ear tagger for individual identification (in many cases, it was not necessary since mice were easily identifiable by white areas on their tails). BCG administration procedures were carried out in the Biosafety Level 2+ (BSL2+) area at the VISAVET Health Surveillance Centre (Complutense University, Madrid). Thirty days after immunization, animals were moved to the Biosafety Level 3 (BSL3) area at the VISAVET Centre for SARS-CoV-2 infection studies.

### SARS-CoV-2 virus and cell lines

SARS-CoV-2 MAD6 was used for experimental infection assay. Calu 3 cells were prepared to reproduce stocks of SARS-CoV-2 [39]. The cells were incubated at 37 °C under 5% CO_2_ in Eagle’s Minimum Essential Medium (EMEM) with l-glutamine (Merck KGaA, Darmstadt, Germany) and supplemented with 100 IU/mL penicillin, 100 μg/mL streptomycin, and 10% fetal bovine serum (FBS) (Merck KGaA, Darmstadt, Germany).

For viral growth in Calu-3 cells, a multiplicity of infection (MOI) of 0.0001 was used. After the virus was absorbed for one hour at 37 °C, the viral growth media EMEM supplemented with 2% fetal bovine serum was added. The cell lysate and supernatant were harvested after 3 days of incubation at 37 °C with 5% CO_2_.

Vero E6 cells, provided by the Carlos III Health Institute (Madrid, Spain), or ATCC® (Manassas, Virginia, USA), were prepared to titrate SARS-CoV-2 stocks by determining the amount of virus causing cytopathic effects in 50% of tissue culture infectious dose (TCID50/mL). Additionally, this cell line was used to verify the viability of the SARS-CoV-2 inoculum used for infecting the animals.

### BCG preparation

BCG was inoculated in a starter liquid culture with a vial of frozen strain, incubated at 37 °C in aerobiosis for 4 weeks and then inoculated in a liquid culture at 37 °C in aerobiosis for another 4 weeks. At the end of the culture, the growth was collected with a pipette, centrifuged, washed with PBS and given a treatment of physical breakage with glass beads, and diluted with PBS until a homogeneous product was at approximately 1 McFarland [equivalent to approximately 10^6^ colony forming units (CFU)/mL]. Once the inoculum was ready, a plate count was performed to determine the concentration of the prepared inoculum, as well as afterwards the inoculation of BCG in the mice. The final dose for inoculation obtained was 1.3 × 10^5^ CFU in 100 µL at a concentration of 1.3 × 10^6^ CFU/mL.

### Experimental design

**Figure 1:**
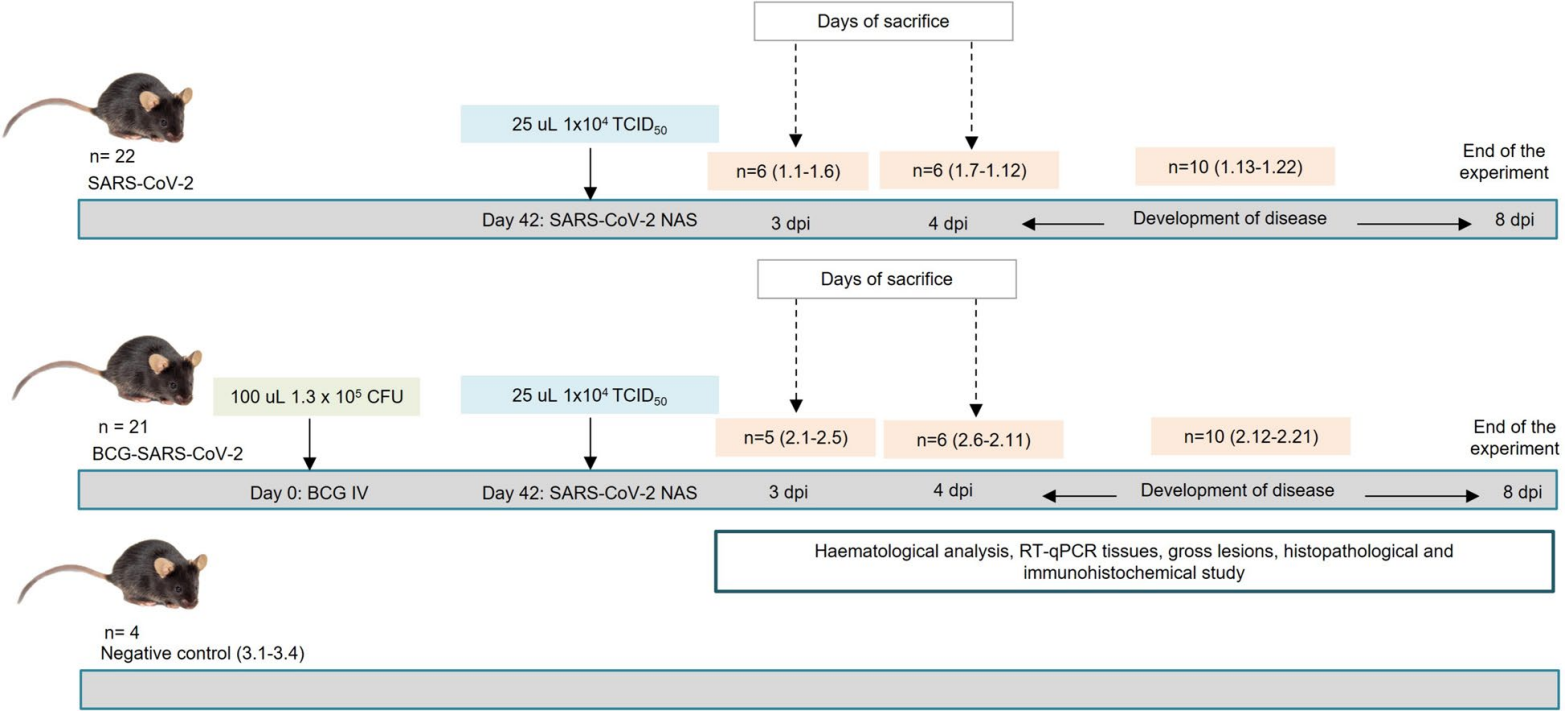
**Experimental design and timeline, depicting the immunization-to-sacrifice period for the three different groups of animals included in the study.** Group 1 (animals challenged with SARS-CoV-2 only, named SARS-CoV-2, *n* = 22; 13 females and 9 males), Group 2 (animals intravenously administered BCG and challenged with SARS-CoV-2, named BCG-SARS-CoV-2, *n* = 21; 12 females and 9 males), Group 3 (negative control animals which were neither BCG-stimulated nor SARS-CoV-2 challenged, named control, *n* = 4; 2 females and 2 males).

Animals were divided into 3 different experimental groups: Group 1, “only challenged with SARS-CoV-2” (SARS-CoV-2, *n* = 22; 13 females and 9 males); Group 2, “BCG-stimulated and SARS-CoV-2 challenged” (BCG-SARS-CoV-2, *n* = 21; 12 females and 9 males); Group 3 “non-stimulated, nor challenged” (negative control animals, *n* = 4; 2 females and 2 males) (Figure 1, Additional file 1). Groups 1 and 2 were divided into two subgroups follow-up duration: (i) animals sacrificed at 3–4 days post-infection (dpi), (ii) animals that developed COVID-19 and were sacrificed when they reached the endpoint criteria described in the following section (5, 6, 7 dpi), or animals left until the 8 dpi (end of the experiment) that did not develop COVID-19 or did not reach endpoint criteria.

### BCG administration, SARS-CoV-2 infection, and sampling

BCG was intravenously (IV) administered using a syringe with a 30 G needle through the caudal vein of the tail (group 2, Figure 1). A restrainer was used to immobilize the mouse while administering BCG. The tail was pre-warmed slightly using a heat lamp for enhanced vasodilation. Animals were challenged with the SARS-CoV-2 virus 6 weeks after the intravenous BCG administration (Figure 1).

For infection, the 43 mice (groups 1 and 2) were sedated with xylazine (20 mg/mL) at a dose of 2 mg/kg and ketamine 100 mg/mL at a dose of 20 mg/kg intraperitoneally (IP). Afterwards, animals were inoculated intranasally (NAS) with SARS-CoV-2 at a dosage of 1 × 10^4^ TCID_50_ per mouse. The total volume inoculated was of 25 μL alternating nostrils in volumetric fractions of 5 μL (Figure 1).

To confirm that animals were successfully infected (groups 1 and 2), oropharyngeal swab samples were collected at 2 dpi [DeltaSwab® Virus with viral transport media (MTV) (Deltalab S.L, Cataluña, Spain)]. In addition, a COVID19 dtec RT-qPCR Test (Genetic PCR Solutions™, Alicante, Spain) test was carried out to quantify the infection inoculum with a result of 1.94 × 10^6^ copies/µL.

Following the challenge, animals were weighed and monitored daily for clinical signs. A clinical scoring table (Table 1) was prepared and utilized to document each clinical sign on a scale from 0 to 2. The animals were sacrificed upon reaching a cumulative clinical score (sum of scores for each evaluated clinical sign) of 4 or a loss of weight higher than 20%. Once the designated sacrifice days were reached (3 and 4 dpi), or endpoint criteria was fulfilled, blood samples in heparin were obtained from the animals after sedation IP with xylazine (20 mg/mL) at a dose of 4 mg/kg and ketamine (100 mg/mL) at a dose of 40 mg/kg, before sacrifice. The animals that did not meet the endpoint criteria were euthanized on day 8 post-infection, marking the end of the experiment. Subsequently, a comprehensive necropsy was conducted on the animals. In this study, brain, lung, trachea, and nasal turbinates samples were collected in AllProtect Tissue Reagent (Qiagen, Venlo, Netherlands) for viral RNA detection. Brain and lung tissues were also collected in 10% neutral formalin for histopathology and IHC studies.

**Table 1:** **Score of the different clinical signs analyzed throughout the experience since the infection of the animals**

Clinical score	0	1	2
Loss of weight	None/slight (0–10%)	Moderate (10–20%)	Severe (> 20%)
Hair appearance	Undamaged	Slightly tousled	Bristly
Level of activity	Normal	Reduced activity	Inactive
Eye closing	Normal eyes	Slightly bent	Totally closed
Respiratory signs	Normal	Slightly increased	Tachypnoea, dyspnoea or abdominal tightening
Neurological signs	None	Depression, bending posture, difficulty in walking	Tremors or convulsions

### SARS-CoV-2 RNA extraction and reverse transcription-quantitative PCR (RT-qPCR)

AllPrep® DNA/RNA/Protein Mini Kit (Qiagen, Venlo, Netherlands) was used for RNA extraction of tissues according to the manufacturer’s instructions. In addition, the detection and quantification of SARS-CoV-2 loads from tissues and swabs was performed using the CoVID19 dtec RT-qPCR Test (Genetic PCR Solutions™, Alicante, Spain).

### Measurements of inflammatory and coagulation markers

Complete heparinized blood was centrifugated and plasma was stored at −80 °C until the analyses of the following biomarkers: CRP (C-reactive protein) (Mouse CRP ELISA Kit. Invitrogen, Massachusetts, USA), ferritin (Mouse Ferritin ELISA kit. Crystal Chem, Zaandam, Netherlands), D-dimer [Mouse D2D (D-Dimer) ELISA Kit. FineTest®, Boulder, USA] and iNOS (nitric oxide synthase) (iNOS ELISA Kit. MyBioSource, San Diego, USA).

Proinflammatory cytokines as interleukin 1β (IL-1β), interleukin 6 (IL-6), tumour necrosis factor alpha (TNF-α), interferon gamma (IFN-γ), and anti-inflammatory as interleukin 1 receptor antagonist (IL-1ra) and transforming growth factor beta-1 (TGF-β1) were measured with the Ella™ Automated Immunoassay System (ProteinSimple, Abingdon, Belgium).

### Histopathological and immunohistochemical evaluation

The lung and brain were fixed in 10% neutral formalin for 48 h. The samples were automatically processed (Citadel 2000 Tissue Processor, Thermo Fisher Scientific, Waltham, MA, USA) and embedded in paraffin (HistoStar Embedding Workstation, Thermo Fisher Scientific). Five consecutive sections of 4 µm thickness were obtained for each case using a microtome (FinesseMe+, Thermo Fisher Scientific). One section was stained with haematoxylin–eosin (HE) (Gemini AS Automated Slide Stainer, Thermo Fisher Scientific) and the following four sections were placed in positively charged glass slides and used for further immunohistochemical studies.

The paraffin sections placed in positively charged glass slides were deparaffinised in xylene and rehydrated. This step was carried out by the Epredia PT module Deparaffin and Heat Induced Epitope Retrieval (HIER) (Thermo Fisher Scientific). Endogenous peroxidase was blocked by immersing the samples in a 3% hydrogen peroxide in methanol solution (Panreac Química S.L.U, Spain) for 15 min. Then, the samples were incubated with 2.5% Normal Horse Serum for blocking (RTU) for 1 h. Afterwards, the slides were subsequently incubated overnight at 4 °C with the primary antibodies detailed in Table 2 (Thermo Fisher Scientific; DAKO, Glostrup, Denmark). Positive and negative controls were included in each batch of slides. For negative controls, the primary antibody was omitted and substituted by tris-buffered saline. After night, secondary antibody was added (ImmPRESS® VR Horse AntiMouse IGG Polymer Kit, Peroxidase; Vector Laboratories, Newark, California, United States) and incubated for 1 h. For the revealing process peroxidase was used (ImmPACT ® NovaRED®Substrate Kit Peroxidase; Vector Laboratories, Newark, California, USA). Finally, samples were mounted (CTM6 Coverslipper, Thermo Fisher Scientific) and evaluated for histopathological alterations under a Leica DM2000 microscope (Leica Microsystems, Wetzlar, 162 Germany).

**Table 2:** **List and details of antibodies used in the immunohistochemical study**

Antibody	Type	Host	Dilution	Company
Anti-SARS-CoV-2	Monoclonal	Mouse	1:100	Thermo Fisher Scientific
Anti-CD3	Polyclonal	Rabbit	1:100	DAKO
Anti-PAX5	Monoclonal	Mouse	Ready to use	DAKO
Anti-Iba-1	Polyclonal	Rabbit	1:50	Thermo Fisher Scientific

Pulmonary histological score was derived from the assessment of 14 parameters across different experimental groups with the objective of characterizing the lung injury severity: pleuritis, septal thickening, peribronchiolar inflammatory cell infiltration, perivascular inflammatory cell infiltration, oedema, desquamative alveolitis, atelectasis, emphysema, type II pneumocytes hyperplasia, bronchiolar hyperplasia, vasculitis, perivascular oedema, haemorrhage, and thrombosis. Each histopathological parameter was evaluated according to a 3-point scale from 0 to 3 indicating: 0 no lesion, 1 mild lesion, 2 moderate lesion, and 3 severe lesions. Mean values and standard deviations of histopathological parameters were calculated for each mouse and for each group of mice.

Histopathological and immunohistochemical scorings were performed in the following brain sections: olfactory bulb, pyriform cortex, septum-striatum, cerebral cortex, hippocampus, thalamus, hypothalamus, mesencephalon, pons, cerebellum, and spinal cord. The parameters assessed were perivascular lymphocytic cuffing, vasculitis, perivascular haemorrhage, thrombosis, shrunken neurons, neuropil spongiosis (cytoplasmic ballooning), white matter tract myelin sheath vacuolation, microglial activation (neuronophagia), oligodendrocyte activation (satellitosis), astroglial activation (astrogliosis). Paxinos and Franklin atlas [40] was consulted for histological identification of the different brain regions. Parameters were scored from 0 to 5 according to their degree of severity, based on the proportion of affected cells/tissue (Table 3). The range score for histopathology goes from 0 to 570 in each animal; one animal was considered affected when it showed a score value higher than 20 (since it was the highest score in negative control animals—group 3—assuming it could be due to artifacts and unspecific lesions). With respect to IHC, SARS-CoV-2 neuronal immunoexpression were scored from 0 to 5 according to their degree of extension, based on the proportion of infected neurons (Table 3); the range score goes from 0 to 55 (Table 3).

**Table 3:** **Evaluation criteria for histopathologic and immunohistochemical scoring (SARS-CoV-2 immunoexpression) according to the amount of nervous tissue affected, classifying the lesions into 5 levels**

	Histopathological score		Immunohistochemical score	
Negative	0% affected tissue/cells	0	Negative	0
Minimal	0–20% affected tissue/cells	1	Focal (single neuron)	1
Mild	20–40% affected tissue/cells	2	Oligofocal (single neuron)	2
Moderate	40–60% affected tissue/cells	3	Focal (neuron aggregates)	3
Marked	60–80% affected tissue/cells	4	Multifocal (neuron aggregates)	4
Severe	80–100% affected tissue/cells	5	Multifocal to coalescing/diffuse (neuron aggregates)	5

### Statistical analysis and graphic creation

Odds ratios and Risk Ratios/Relative risks were performed using MedCalc for Windows, version 22.014 (MedCalc Software, Ostend, Belgium).

Correlation between the detection of SARS-CoV-2 in the lung and brain was estimated with Spearman’s Rho coefficient in IBM SPSS Statistics for Windows, version 287.0. 1. 1 (15) (IBM Corp, Armonk, N.Y, USA). Differences between groups regarding to qualitative variables (proportion of PCR-positive brain samples) were tested using Fisher’s test in SPSS. Differences between groups regarding to quantitative variables (viral loads in organs, histopathological brain and lung lesions, cytokine measurements and hematological inflammation markers) were evaluated using Kruskal–Wallis test and Mann–Whitney test in SPSS. Mann–Whitney *p*-values were adjusted according to FDR (False Discovery Rate). Statistically significant differences in all tests were considered when *p*-value was < 0.05.

**Figure 2:**
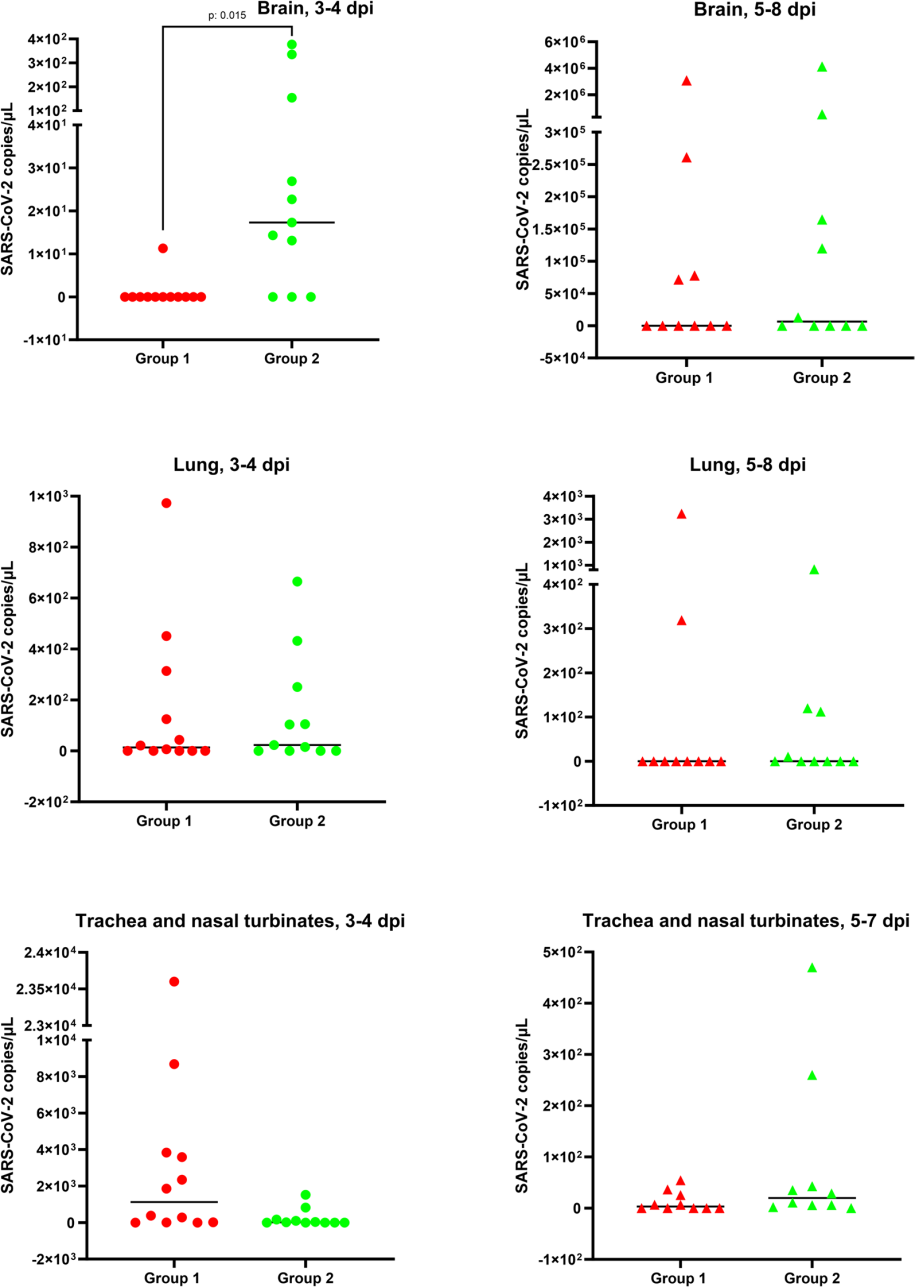
**Plot representation of PCR results for each animal from group 1 (SARS-CoV-2) and group 2 (BCG-SARS-CoV-2).** These groups are separated in two different subgroups: (i) 3–4 dpi and (ii) development of disease. Brain, lung, and trachea/nasal turbinates viral loads (copies/µL of purified RNA) are depicted with red (group 1, dots for 3–4 dpi, triangles 5–8 dpi) and green (group 2, dots for 3–4 dpi, triangles for 5–8 dpi). The SARS-CoV-2 loads (copies/µL) from brain, lung and trachea/nasal turbinates are shown. The limit of detection cutoff for the PCR corresponded to 2 copies/µL. The horizontal bars represent the median of the values for each group.

**Figure 3:**
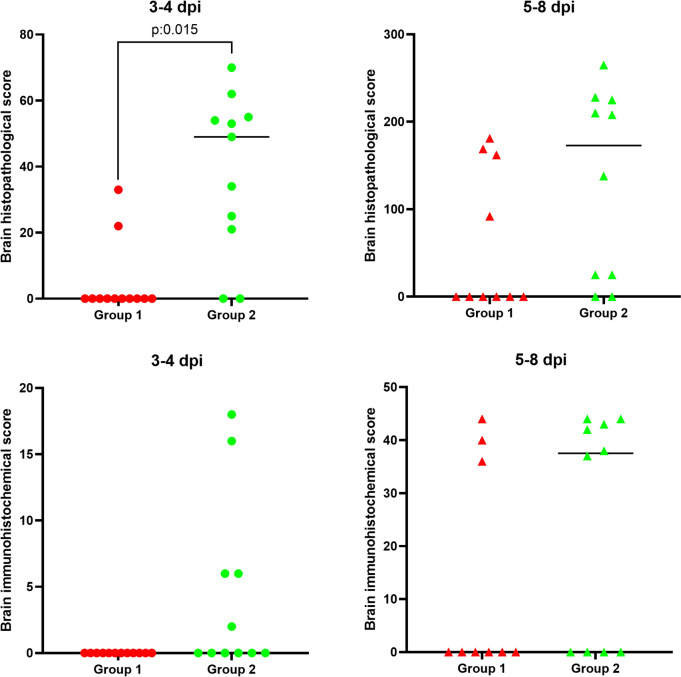
**Plot representation of results for each animal from group 1 (SARS-CoV-2) and group 2 (BCG-SARS-CoV-2).** These groups are separated in two different subgroups: (i) 3–4 dpi and (ii) development of disease. Brain histopathological and immunohistochemical score are depicted with red (group 1, dots for 3–4 dpi, triangles 5–8 dpi) and green (group 2, dots for 3–4 dpi, triangles 5–8 dpi). The horizontal bars represent the median of the values for each group.

Figures 2, 3 and 6 were created using GraphPad Prism 10 software.

ChatGPT was used on some occasions to improve the drafting of the article.

## Results

### BCG-stimulated SARS-CoV-2 challenged animals presented higher viral loads in brain than not stimulated mice

The study of the disease progression as well as the measurement of viral loads are valuable tools to learn about the development of the disease in different experimental groups assessing the influence of BCG stimulation before SARS-CoV-2 infection. The number of PCR-positive animals in brain samples was significantly higher in BCG-SARS-CoV-2 challenged mice (Fisher’s test, *p* = 0.034). In group 1 (SARS-CoV-2), 5 mice out of 22 (22.7%) tested positive in brain RT-qPCR from 5 dpi onward. The animal with the highest brain viral load in this group, was found dead on 7 dpi (Additional file 1). In the BCG-SARS-CoV-2 mice (group 2), 14 out of 21 mice (66.6%) tested positive for brain RT-qPCR, being the highest viral load of an animal euthanized at 6 dpi (2.13). The highest brain viral loads were observed in animals euthanized at 6–7 dpi, coinciding with the highest clinical scores (Figure 2, Additional file 1). No animal euthanized at 8 dpi from any of the groups showed brain viral loads.

Group 2 (BCG-SARS-CoV-2) had significantly higher brain viral loads than group 1 (SARS-CoV-2) (Mann–Whitney test, *p* = 0.042). These differences were focused at 3–4 dpi (*p* = 0.015) while no significant differences were observed at 5–8 dpi (Figure 2).

The RT-qPCR was also performed on lung, nasal turbinate, and trachea samples. In lungs, similar viral loads between groups were obtained (mean copies/µL group 1 = 0.40, SD = 0.50; mean group 2 = 0.52, SD = 0.51). No significant differences were observed either in the RT-qPCR results for lungs between both groups (Mann–Whitney test, *p* = 0.821) (Figure 2). The highest lung viral loads were observed between 3 and 7 dpi, while at 8 dpi no viral loads were detected. A positive correlation was observed between the detection of SARS-CoV-2 in the lung and brain (Spearman’s Rho coefficient = 0.375; *p* = 0.013). Regarding nasal turbinate and trachea samples, viral RNA only persisted in BCG-SARS-CoV-2 animals until 8 dpi (Figure 2).

In group 1 (SARS-CoV-2), a morbidity rate of 40% was observed (4 out of 10 mice presented clinical signs and were sacrificed), while in mice belonging to group 2 (BCG-SARS-CoV-2), the morbidity rate after SARS-CoV-2 infection was 60% (6 out of 10 mice) (OR = 2.25 95% CI = 0.37–13.46). The challenged animals that remained clinically healthy were sacrificed on the last day of the experiment (8 dpi). Animals started to show clinical signs from 5 dpi onwards, with the most observed signs being reduced activity and weight loss (3 animals in group 1 and 6 animals in group 2). At the time of death (5–7 dpi), the median clinical score was of 5 ± 0.87 (Additional file 1).

### BCG stimulation increased the severity of SARS-CoV-2-induced brain lesions

Neuroinvasion and dissemination are key to the fatal development of SARS-CoV-2 in this experimental model [41]. For these reasons, histopathological and IHC studies were carried out. Animals from BCG-SARS-CoV-2 group (group 2) exhibited significantly more severe brain lesions due to SARS-CoV-2 infection in comparison to mice from group 1, as demonstrated by histological analysis (Mann–Whitney test, *p* = 0.001) (Figure 3).

**Figure 4:**
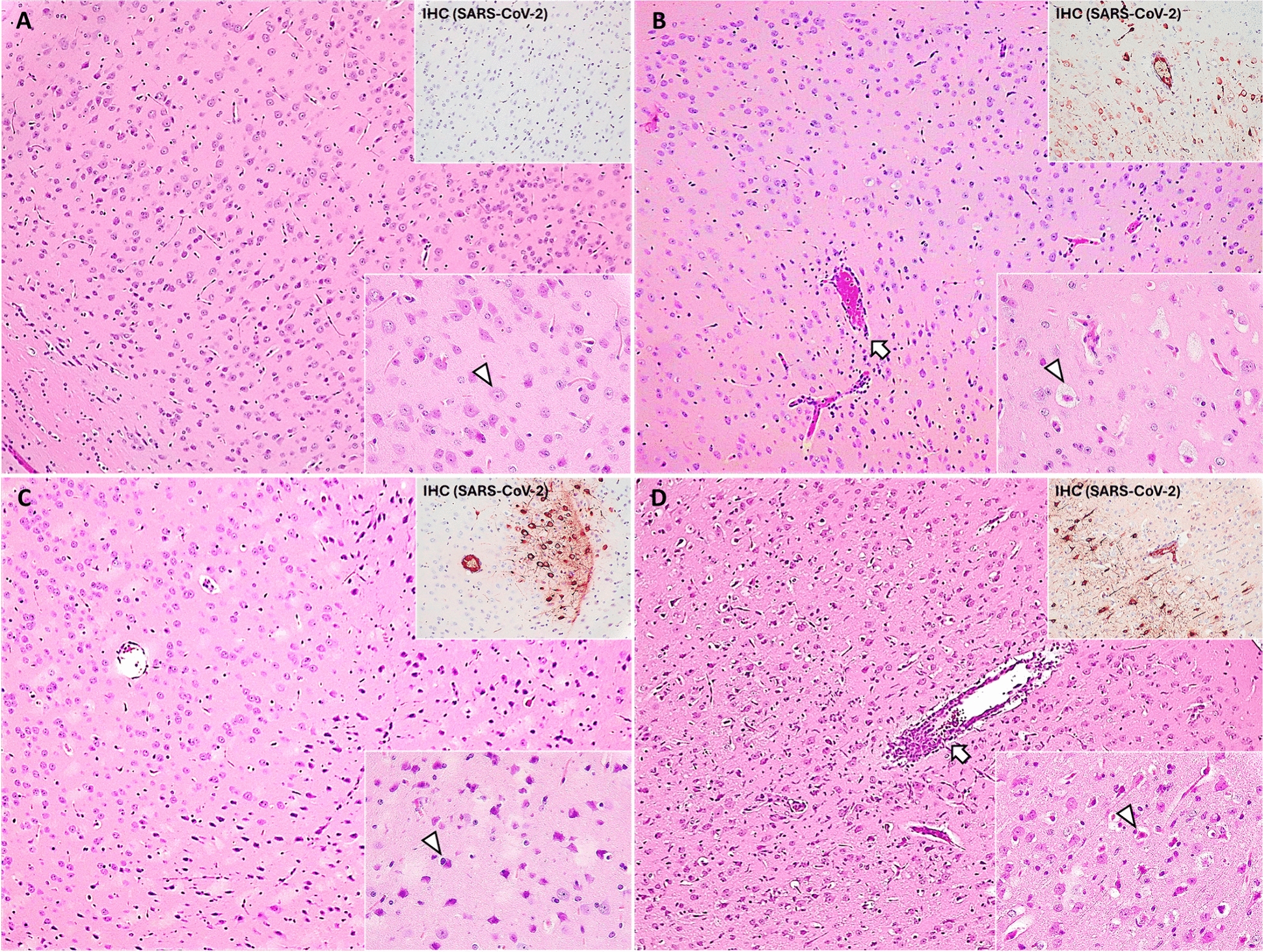
**Microscopic brain lesions and viral distribution in the orbital cortex in group 1 (SARS-CoV-2) and group 2 (BCG-SARS-CoV-2) at 3–4 (A, C) and 5–7 dpi (B, D).** **A** Absence of lesions with a normal glial cell population number; HE, ×10. Lower right inset: non-damaged neuron (arrowhead); HE, ×40. Upper right inset: absence of SARS-CoV-2 immunoexpression; IHC, ×20. **B** Mild to moderate perivascular lymphocytic cuffs (arrow); HE, ×10. Lower right inset: moderate number of neurons with cytoplasmic balloning and shrunken red nuclei (neuronal degeneration—arrowhead); HE, ×40. Upper right inset: neuronal SARS-CoV-2 immunoexpression in cortical layers IV-V; IHC, ×20. **C** Moderate number of perivascular shrunken neurons. HE, ×10. Lower right inset: shrunken basophilic neurons and activated microglia surrounding the soma (arrowhead); HE, ×40. Upper right inset: perivascular neuronal aggregation with SARS-CoV-2 immunoexpression; IHC, ×20. **D** Severe perivascular lymphocytic cuffs (arrow) and increased number of glial cell population. HE, ×10. Lower right inset: severe number of neurons with neuronal degeneration and activated microglia surrounding the soma (arrowhead); HE, ×40. Upper right inset: neuronal SARS-CoV-2 immunoexpression in cortical layers IV-V; IHC, ×20.

Meningoencephalitis was characterized by leptomeningeal mononuclear infiltrates and perivascular cuffs (T lymphocytes), with increased glial cell population, mainly microglia and astrocytes. No relevant inflammatory brain lesions were observed at 3 dpi, except for one BCG-SARS-CoV-2 case (group 2, 1/5 cases; histopathological score (HS) range = 8–31), At 4 dpi, mild non-suppurative meningoencephalitis was infrequently observed in both BCG-SARS-CoV-2 (group 2, 2/6 cases; HS range = 12–41) and SARS-CoV-2 (group 1, 1/6 cases; HS range = 3–19) groups. A notable shift in lesion severity occurred from 5 dpi onwards, affecting almost the entire brain (except for the cerebellum), and cervical spinal cord. At 6–7 dpi, non-suppurative meningoencephalitis reached its maximum severity, with a statistically significant higher histopathological score in BCG-SARS-CoV-2 group (5/5 cases; HS range = 107–143) compared to SARS-CoV-2 group (3/3 cases; HS range = 95–107) (Mann–Whitney test, *p* = 0.036) (Figure 4).

Vascular alterations included lymphoplasmacytic infiltration into the vascular wall (vasculitis), occasional obstruction due to degenerated erythrocytes attached to the vascular wall, and perivascular microhaemorrhages in both grey and white matter, as well as in the subarachnoid space. Minimal vascular alterations were observed at 3 dpi in both BCG-SARS-CoV-2 (group 2, 3/5 cases HS range = 1–11) and SARS-CoV-2 groups (group 1, 1/6 cases; HS range = 1–6). At 4 dpi, the BCG-SARS-CoV-2 group (4/6 cases; HS range = 2–22) in terms of mild vasculitis and microhaemorrhages showed a relative risk of 4.6 (HE 95% CI = 0.69–31.22) compared to group 1 (1/6 cases; HS range = 1–5) At 5 dpi, the severity of vasculitis and haemorrhages increased, reaching their maximum severity at 6–7 dpi. Hyaline thrombi deposition at the vascular wall was observed to a lesser extent. These vascular alterations were more severe in BCG-SARS-CoV-2 animals (5 cases; HS range = 29–55) than in SARS-CoV-2 animals (3 cases; HS range = 26–34) (Mann–Whitney test, *p* = 0.053).

At 3–4 dpi, mild neuronal alterations were observed in BCG-SARS-CoV-2 group (7/11 cases; HS range = 3–18) and minimally in SARS-CoV-2 group (2/12 cases; HS range = 4–6) (Figure 4). These alterations included the presence of red shrunken neurons, adjacent to areas of increased glial cell population and vascular damage. At 4 dpi, swollen neurons with cytoplasmic balloning and pyknotic and eccentric nuclei (neuronal degeneration) began to appear in both groups. These findings were prevalent in several brain regions and the spinal cord at 5 dpi. The maximum expression of lesions was observed at 6–7 dpi, with extensive neuronal degeneration. Bilateral and symmetrical myelin sheath vacuolation was displayed in the white matter tracts, affecting major myelin-rich areas. Neuronal alterations were more frequent and significantly more severe in BCG-SARS-CoV-2 animals (5 cases; HS range = 54–80) than in SARS-CoV-2 animals (3 cases; HS range = 40–47) (Mann–Whitney test, *p* = 0.024) (Figure 4). Animals sacrificed at 8 dpi had no significant lesions. Additional file 2 shows more detailed information concerning histopathological score results.

### Increased neuroinvasion and interneuronal dissemination of SARS-CoV-2 antigen in BCG-stimulated animals

BCG-SARS-CoV-2 animals (group 2) showed a relative risk of developing brain lesions due to SARS-CoV-2 at 3–4 dpi of 4.9 (HE 95% CI = 1.34–17.93) and 11.9 (IHC 95% CI = 0.73 to 193.38) times higher compared to animals from group 1. At days 5–8, BCG-SARS-CoV-2 animals (group 2) showed a relative risk of developing brain lesions of 2 (HE 95%IC = 0.88–4.54) and 1.5 (IHC 95% IC = 0.60–3.73) times higher compared to animals from group 1.

At 3–4 dpi, group 2 showed mild neuronal and microglial immunoexpression against SARS-CoV-2 in 5 out of 11 cases, while group 1 displayed none in 12 cases. Notably, at 3 dpi, group 2 [2 out of 5 cases; IHC (IS) range = 2–6] exhibited oligofocal immunoexpression in the olfactory bulb, orbital cortex, pyriform cortex, olfactory tubercle, the nucleus of the lateral olfactory tract and the medio dorsal thalamic nucleus. At 4 dpi, group 2 (3 out of 6 cases; IS range = 6–18), showed more widespread viral immunoexpression with infected neuronal aggregates in the olfactory pathway areas. In addition, infected single neurons began to appear in frontal cortex, as well as periventricular nucleus of the septum/striatum, thalamus, hypothalamus, mesencephalon, pons and spinal cord.

At 5 dpi, both BCG-SARS-CoV-2 (Group 2, 1 out of 1 case; IS range = 42) and SARS-CoV-2 groups (Group 1, 1 out of 1 case; IS range = 38) exhibited an evident increase of viral spreading. Multifocal to coalescent/diffuse neuronal immunoexpression was observed, noting the onset of hippocampal infection. At 6–7 dpi, BCG-SARS-CoV-2 (Group 2, 5 out of 5 cases; IS range = 37–44) and SARS-CoV-2 (Group 1, 3 out of 3 cases; IS range = 36–44) animals displayed their maximum peak antigen presence. Despite the virus dissemination being highly similar in both groups, there was an apparent reduction in the number of neurons exhibiting positive immunoreactivity in the BCG-SARS-CoV-2 group, potentially attributed to an increase in the number of dead neurons. Cerebellar neurons showed no infection, but microglial immunoexpression adjacent to perivascular cuffs was observed in the cerebellar white matter. Animals sacrificed at 8 dpi did not display IHC antigen expression. Overall, a significantly higher total histopathological and IHC score were observed in BCG-SARS-CoV-2 animals (total HS = 1793; total IS = 296) than SARS-CoV-2 animals (total HS = 825; total IS = 158) (Mann–Whitney test HS *p* = 0.002; Mann–Whitney test IS, *p* = 0.006). Additional file 3 shows more detailed information concerning histopathological score results. Detailed histopathological and IHC images comparing both groups are shown in Additional file 4.

### Severity of lung lesions was slightly higher in BCG-stimulated SARS-CoV-2 challenged mice

**Figure 5:**
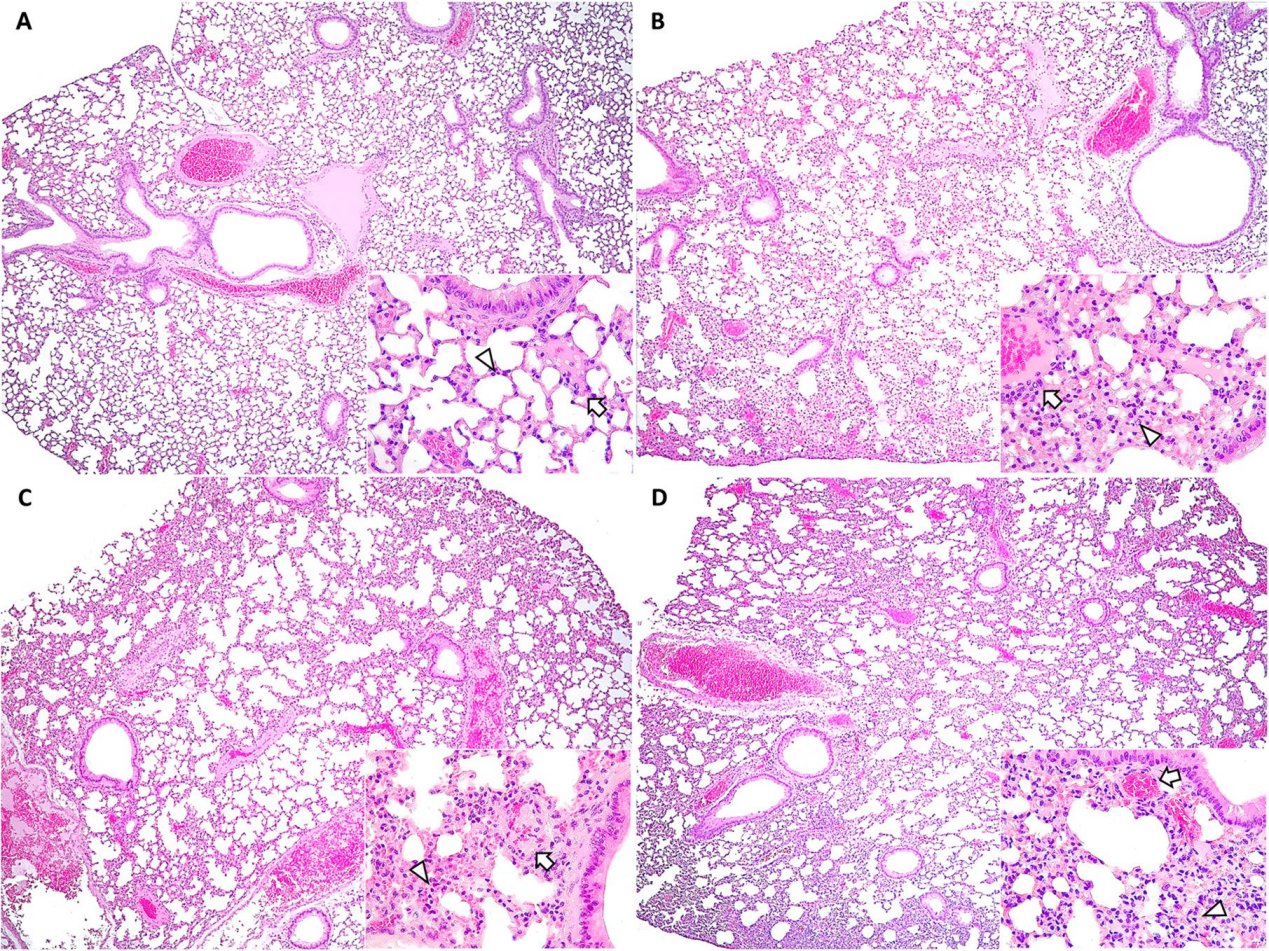
**Microscopic lung lesions in mice from: group 1 (SARS-CoV-2) at 3–4 dpi (A) and 5–8 dpi (B); and group 2 (BCG-SARS-CoV-2) at 3–4 dpi (C) and 5–8 dpi (D).** **A** Absence of lesions; HE, ×10. Lower right inset: normal alveolar septum thickness (arrowhead) and no perivascular inflammation (arrow); HE, ×40. **B** Mild to moderate and patchy thickening of alveolar septum; HE, ×10. Lower right inset: moderate interstitial pneumonia (arrowhead) and perivascular lymphocytic inflammation (perivascular cuffs—arrow); HE, ×40. **C** Mild to moderate patchy thickening of alveolar septum; HE, ×10. Lower right inset: mild to moderate interstitial pneumonia (arrowhead) and vascular thrombosis (arrow); H&E, ×40. **D** Moderate thickening of alveolar septum thickness; HE, ×10. Lower right inset: moderate interstitial pneumonia (arrowhead) and mild perivascular cuffs (arrow); HE, ×40.

Lungs are one of the most affected organs by SARS-CoV-2 infection, so a histological study was carried out to determine differences between BCG-stimulated and non-stimulated challenged animals. ACE2-expressing alveolar type 2 pneumocytes and goblet cells are the main targets, among others, of SARS-CoV-2 in human lungs [42]. The primary finding in this study was bronchointerstitial pneumonia, characterized by infiltration of mononuclear cells (mainly macrophages and T lymphocytes) and occasional neutrophils around bronchioles and blood vessels. Group 2 exhibited a significantly more moderate pneumonia than group 1 (Mann–Whitney test, *p* = 0.007), with this difference being significantly more pronounced at 5–8 dpi (Mann–Whitney test, *p* = 0.028) (Figure 5). Animals in group 2 exhibited a significantly higher frequency of inflammatory cell infiltration in the alveolar interstitium (Mann–Whitney test, *p* = 0.017) and pleura (Mann–Whitney test, *p* = 0.009), with those inflammatory foci mainly consisting of mononuclear cells (including abundant foamy macrophages).

Vascular and oedematous lesions were frequent, being group 2 the one exhibiting the highest occurrences of vascular thrombosis and perivascular oedema at 3–4 dpi even though observed differences in animals sacrificed in these dpi were not statistically significant (Mann–Whitney test, *p* = 0.630; *p* = 0.551 respectively). In addition, type II pneumocyte hyperplasia and bronchiolar epithelium hyperplasia were more intense in the group 2, mainly at 5–8 dpi even though no significant differences were detected in these days post-infection (Mann–Whitney test, *p* = 0.442; *p* = 0.083 respectively). On the other hand, atelectasis was significantly more intense in group 2 at 5–8 dpi (Mann–Whitney test, *p* = 0.038). Detailed histopathological images of the BCG- SARS-CoV-2 group at 5–8 dpi are presented in Additional file 5.

The Additional file 6 shows the immunohistochemical assessment of cellular markers to detect the lymphocytic phenotype and the presence of macrophages in the inflammatory reaction observed in both brain and lungs.

### BCG stimulation modified plasma inflammatory and coagulation biomarkers response at different post-immunomodulation days

**Figure 6:**
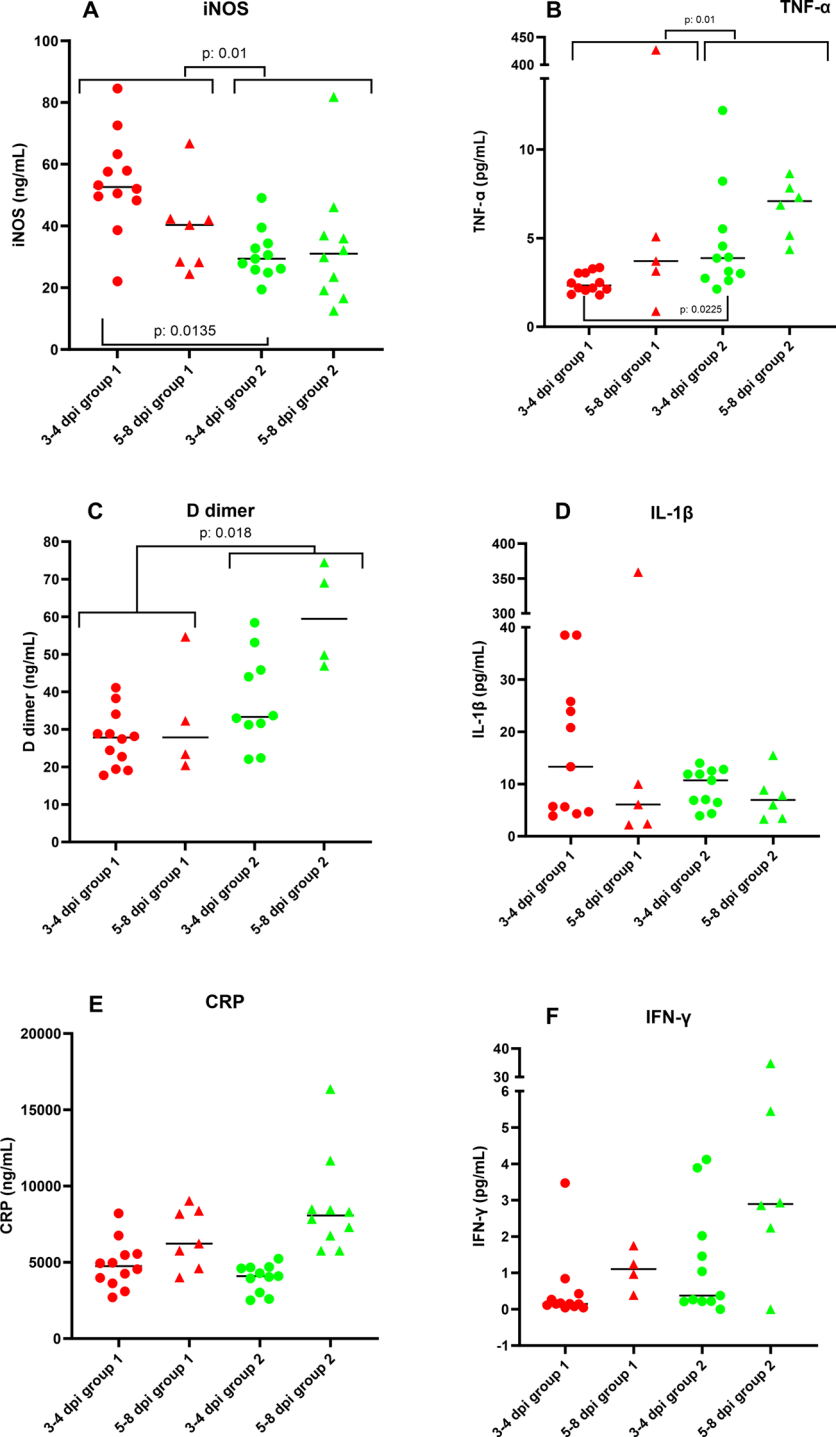
**Plot representation of the results obtained for iNOS (A), CRP (C), D-dimer (E) in ng/mL, and the cytokines TNF-α (B), IL1-β (D) and IFN-γ (F) (pg/mL) for each group.** These groups are separated in two different subgroups: 3–4 dpi (red dots for group 1 and green dots for group 2) and development of disease (5–8 dpi) (red triangles for group 1 and green triangles for group 2). Horizontal bars represent the median of the values for each group. Missing results are due to the limited plasma volume, which did not allow the analysis of all parameters in some animals.

Variations and abnormalities in plasma inflammatory and coagulation biomarkers have been noted at different stages of the disease in human patients [43]. For this reason, an evaluation of these parameters was carried out to assess if there were any differences between experimental groups 1 and 2. Results for plasma inflammatory and coagulation biomarkers for each animal are shown in Table 4 with median values for experimental groups 1 and 2. These parameters were compared between experimental groups in general and in two different periods post-infection: 3–4 dpi and development of disease (5–8 dpi). There were significant differences in the D-dimer values between both groups, presenting higher values the BCG-SARS-CoV-2 group (Mann–Whitney test, *p* = 0.019) (Figure 6, Table 4). In contrast, iNOS values were higher in SARS-CoV-2 animals (group 1) (Figure 6, Table 4) (Mann–Whitney test, *p* = 0.01) in general and, comparing both groups at 3–4 dpi (Mann–Whitney test, *p* = 0.013) (Figure 6). Animals from BCG-SARS-CoV-2 group showed higher values for TNF-α, compared to animals from group 1 (Mann–Whitney test, *p* = 0.01). These significant differences were also observed on 3–4 dpi (*p* = 0.022) (Table 4, Figure 6).

**Table 4:** **Median values and standard deviation for D-dimer, CRP (C-reactive protein), ferritin, iNOS (nitric oxide synthase) as well as for cytokines [interleukin 1β (IL-1β), interleukin 1 receptor antagonist (IL-1ra), interleukin 6 (IL-6), tumour necrosis factor alpha (TNF-α), interferon gamma (IFN-γ), and transforming growth factor beta-1 (TGF-β1)] for group 1 (SARS-CoV-2) and 2 (BCG-SARS-CoV-2) in the moment of the sacrifice**

	All			3–4 dpi			Development (5–8 dpi)		
	Group 1	Group 2	*p-*value	Group 1	Group 2	*p-*value	Group 1	Group 2	*p-*value
D-Dimer	27.46 ± 11.29	33.68 ± 47.33	**0.019**	27.81 ± 7.45	33.68 ± 59.08	0.09	28.90 ± 13.42	57.72 ± 13.75	0.515
C-reactive protein	4974 ± 1869.15	5234 ± 3278.97	0.094	4747 ± 1542.37	4096 ± 827.52	0.621	6073.65 ± 1961.56	7490.39 ± 1795.22	1
Ferritin	1258.20 ± 391.65	1038.48 ± 515.15	0.107	1107.62 ± 301.28	1046.96 ± 688.12	1	1416.33 ± 466.70	1059.17 ± 624.00	0.515
iNOS	49.59 ± 16.62	29.91 ± 14.55	**0.01**	52.62 ± 15.71	29.40 ± 8.04	**0.013**	35.26 ± 14.32	25.79 ± 19.87	0.869
IL1-β	8.035 ± 87.19	7.85 ± 3.93	0.821	20.8 ± 13.58	10.7 ± 3.64	0.652	4.23 ± 3.68	6.94 ± 4.52	1
IL-ra	220 ± 149.02	149 ± 79.69	0.165	223 ± 87.68	124 ± 90.63	0.0956	165 ± 265.04	190.5 ± 59.68	1
IL-6	2.32 ± 78.69	3.78 ± 55.98	0.941	2.64 ± 108.05	3.78 ± 7.42	0.943	4.13 ± 14.59	5.46 ± 90.55	1
TNF-α	2.5 ± 102.92	4.55 ± 2.73	**0.01**	2.34 ± 0.55	3.87 ± 3.01	**0.022**	3.71 ± 189.53	7.09 ± 1.63	0.515
IFN-γ	0.219 ± 0.90	1.25 ± 8.47	0.096	0.15 ± 0.96	0.38 ± 1.50	0.0956	1.10 ± 0.57	2.86 ± 14.51	0.66
TGF-β1	4999 ± 4638.84	4010 ± 4247.79	0.941	3345 ± 4847.62	4010 ± 4396.57	0.757	5678.5 ± 4550.38	3822 ± 4001.07	0.515

Statistically significant *p*-values are highlighted in bold. Missing results are due to the limited plasma volume, which did not allow the analysis of all parameters in some animals.

## Discussion

Following the COVID-19 pandemic, a question arose about the potential benefits of BCG vaccination in enhancing the immune response against SARS-CoV-2 [15]. Controversial results have emerged from both epidemiological and experimental investigations [26, 30, 34–36, 44]. For this reason, more studies are needed to clarify and test the effect of BCG prior to SARS-CoV-2 infection [27, 45].

In this study, it was investigated whether prior BCG stimulation might affect clinical signs, morbidity, mortality, plasma inflammatory/coagulation biomarkers, and cytokine values following SARS-CoV-2 experimental infection. In addition, viral loads (nasal turbinates/trachea, lung, and brain) by RT-qPCR and histopathology (lung and brain) were assessed. Immunohistochemical analysis was also carried out to confirm invasion and spread of the virus in brain areas. It is important to note that this experimental model is associated with an increased lethality due to SARS-CoV-2 infection and higher neurodissemination compared to humans. In fact, some limitations of this animal model are the risk of ectopic hACE2 expression which changes the cellular tropism of the virus, as well as non-physiological levels of hACE2 expression [46]. Additionally, this model lacks the ability to induce comorbidities as seen in humans [47]. Even K18-hACE2 model does not fully replicate the COVID-19 signs observed in humans, it serves as a valuable model for studying brain invasion and dissemination [48]. The experimental model is one of the factors that could lead to differences in BCG effect yet experimental studies in other animal models such as golden Syrian hamster [37], report the IV BCG protection to an even higher dose of SARS-CoV-2 than the one used in this study.

Our results indicate that BCG stimulation did not protect K18-hACE2 mice against SARS-CoV-2 in the assayed conditions; in fact, there was a tendency for increased severity, in contrast to previous studies [34, 35, 37]. In group 1 (SARS-CoV-2), only 4 out of 10 mice presented clinical signs, while in the BCG-SARS-CoV-2 mice (group 2), 6 out of 10 showed signs of illness. Reduced activity and weight loss were the most common clinical signs observed in sick mice, regardless of their experimental group, as previously observed [49]. This contrasts with the study by Hilligan et al. [30], where BCG-SARS-CoV-2 animals showed no clinical signs, and the study by Kaufmann et al. [35], in which no differences were observed between the two experimental groups.

Regarding the presence of SARS-CoV-2 in brain tissue, the number of PCR-positive samples was significantly higher in BCG-SARS-CoV-2 challenged mice. The maximum peak was observed at 6–7 dpi, which is in accordance with Carossino et al. [41] and differs from Hilligan et al. [30], a study in which the highest virus levels were observed at 5 dpi, but they only measured viral loads levels at 3 and 5 dpi. In this study, elevated viral loads as well as higher neuroinvasion was detected in the BCG-SARS-CoV-2 group when comparing to the SARS-CoV-2 group, with a significant difference observed at early stages (3–4 dpi). These results contrast with other comparative studies between these two experimental groups in which the same challenge dose was used for infection and no significant differences were observed [30].

Neuroinvasion, the primary cause of SARS-CoV-2 lethality in K18-hACE2 mice [41], has been well-documented in both experimental mouse models [31, 32, 48] and humans [50, 51]. This study further enhances our understanding of SARS-CoV-2 neuroinvasion and pathogenic mechanisms in this experimental model.

The exact pathways of virus entry into neuronal tissues remain controversial. One plausible hypothesis is the olfactory pathway, as SARS-CoV-2 antigens and RNA have been detected in sustentacular cells and Bowman glands [4]. Indeed, in k18-hACE mice ACE2 receptors are notably elevated in the olfactory bulb and in pericytes and endothelial cells [52]. It has also been demonstrated that SARS-CoV-2 is able to infect and cross through microvascular endothelial cells (BMECs), present in the BBB (Blood Brain Barrier) [53]. However, other molecules, like neuropilin-1 (NRP1), especially in olfactory neuroepithelial cells, may also facilitate the viral entry [54]. The present study chronologically examined the most affected areas by SARS-CoV-2 in CNS (central nervous system), revealing virus presence in olfactory-related regions (olfactory bulb, olfactory tubercle, nucleus of the lateral olfactory tract), thalamus, and orbital cortex at 3 dpi. The presence of SARS-CoV-2 in the olfactory bulb has also been appreciated in other studies in k18-hACE2 [32, 33] or humans [55], though the contrary has also been stated [56, 57]. The presence of the virus in this location could explain the altered olfactory reception in k18-hACE2 mice [33], as this information is sent from the olfactory bulb to the orbitofrontal cortex via the dorsal medial nucleus of the thalamus [58], through neuron-to-neuron axonal transport [59]. However, this hypothesis is less likely in humans, as it is suggested that anosmia is primarily caused by a disruption in the olfactory epithelium, where SARS-CoV-2 infects non-neuronal cell types, leading to tissue damage and inflammation [60]. From 4 dpi onwards, infected neuronal aggregations suggest trans-synaptic viral spread between neurons [32], often adjacent to affected blood vessels. In BCG-SARS-CoV-2 mice, at 4 dpi, infection of the subarachnoid space from the olfactory bulb resulted in perivascular meningitis. This space contains crucial large brain blood vessels, potentially affecting BBB permeability. By 5 dpi, high immunoexpression was observed in ventricular areas, vulnerable regions to BBB disruption, alongside reduced immunoexpression in the olfactory bulb or pyriform cortex. In later stages (5–7 dpi), multifocal to diffuse infected areas closely related to vascular and ventricular structures were observed. These findings suggest that after the initial entry via the olfactory pathway, the BBB is altered, promoting hematogenous dissemination, with a possible role of leukocytes carrying SARS-CoV-2 in this process [32].

SARS-CoV-2 was only found in vessels and microglia in the white matter near the pons, raising questions about whether the absence of viral particles found in cerebellar neurons in this study would be due to receptor scarcity [32] or animals dying before virus reaches grey matter area of the cerebellum. Further studies would be necessary to elucidate the viral chronology and presence in these structures.

BCG-SARS-CoV-2 mice presented more severe brain lesions than SARS-CoV-2 animals, consisting of increased glial cells (microglia mainly), T lymphocyte infiltration, and neurodegeneration. In cases of CNS insult, peripheral proinflammatory cytokines such as IL-1, TNF-α, IL-6, IL-12 as well as thrombin, fibrinogen and plasmin concentrations elevate after SARS-CoV-2 infection [61]. This event can cause hypoxia [61], microgliosis and astrogliosis [62] followed by a BBB break down allowing SARS-CoV-2 entrance in the CNS [61, 63], which could be potentially exacerbated in BCG-SARS-CoV-2 animals. The TNF-α, one of the major mediators of the neuroinflammation associated with neurodegeneration, was increased in plasma in BCG-SARS-CoV-2 group probably because of an enhanced Th1 response in these animals [64, 65]. Higher D-dimer levels were also observed in this group, linked to the risk of venous thromboembolism [66]. Similarly, vascular thrombosis was observed in lungs with a slight increase in BCG-SARS-CoV-2 animals. Significantly higher levels of iNOS were observed in the SARS-CoV-2 mice (group 1), which could have possibly played a role in the lower thrombosis events encountered in these animals [67, 68].

The respiratory pathway plays a pivotal role in the SARS-CoV-2 infection process, which is the main reason for its rapid transmission [69]. In our study, both experimental groups (BCG-SARS-CoV-2 and SARS-CoV-2) exhibited similar viral loads in the lungs in contrast with other studies [34, 37] that used different experimental model, dose or strain respectively, obtaining lower viral loads in lungs on BCG inoculated animals. In our study, inflammatory infiltrates were mainly composed of macrophages and lymphocytes, a phenomenon observed by other researchers [42]. Nevertheless, inflammatory lesions were slightly increased in BCG-SARS-CoV-2 mice (group 2) compared to SARS-CoV-2 group. These results contrast with previous studies suggesting that BCG curtails SARS-CoV-2 induced disease severity and lung inflammation [30, 34].

Given all the aforementioned and the discrepancies of this study compared to others that have been carried out to assess the efficacy of BCG against SARS-CoV-2 [30, 34, 37], we posit the possibility that these differences may be attributed to the SARS-CoV-2 variant, the infection dose and the experimental model as already mentioned, as well as the different BCG strain, route and time of BCG administration. Concerning the BCG strain, variations in protective effects against tuberculosis have been demonstrated [70], which could impact the ability to induce protection against unrelated pathogens [71]. In our study, we used the Danish CCUG 27863 strain, while previous studies utilized the Tokyo [34], Pasteur [72], and TICE [35] strains. At this point, it should be considered that previous experiments of our group have demonstrated that animals immunized with the Danish CCUG 27863 strain did not show any vascular or inflammatory changes in brain tissue (data not shown). Thus, we discard that neurological lesions are due to the direct action of BCG. The route of BCG administration may also be relevant, as some studies have assessed the route’s importance in inducing trained immunity [73]. Previous studies in k18-hACE2 mice model used the subcutaneous route for BCG administration and this route failed to protect against SARS-CoV-2 [30, 34, 35]. In fact, BCG intravenous inoculation has been suggested as the only route capable of inducing trained immunity [30, 34] against SARS-CoV-2. However, in our study, even with BCG intravenous inoculation, protection against SARS-CoV-2 was not achieved. SARS-CoV-2 variants have been associated with different neuroinvasive patterns in K18-hACE2 mice [74], potentially explaining different levels of BCG-mediated trained immunity protection against this virus in other studies challenging with different variants [34, 35, 72].

In conclusion, the broad benefits of trained immunity induced by BCG enhancing the host’s immune response against heterologous pathogens [75] and even enhance antitumour immune response [76] have been widely demonstrated. Nevertheless, potential detrimental impact on inflammatory disease has also been suggested in mice [77]. Our results suggest that the dysregulation of innate immune system during SARS-CoV-2 infection [27] could be exacerbated in BCG-stimulated-challenged animals which may lead to a potential harmful impact on the neurological system. This research might constitute a starting point for discussions on the risk of potential adverse outcomes due to the non-specific effects of BCG vaccination.

### Supplementary Information

**Additional file 1. Representation of clinical scores of infected animals at the day of sacrifice and viral loads (brain, lung, trachea/nasal turbinates) from both groups.** *The clinical score from this animal could not be recorded because it was found dead at 7 dpi. **These scores correspond to the day on which the animals were euthanized which coincided with the day of the highest clinical score.

**Additional file 2. Detailed histological score of the animals from both groups for the different brain sections.** Sections analysed are: olfactory bulb, pyriform cortex, septum-striatum, cerebral cortex, hippocampus, thalamus, hypothalamus, caudal mesencephalon, pons, cerebellum, and spinal cord scoring each lesion from 0 to 5.

**Additional file 3. Detailed neuronal immunoexpresion score of the animals from both groups for the different brain sections.** Immunoexpresion was scored from 0 to 5 according to their degree of extension, based on the proportion of affected neurons.

**Additional file 4. Brain lesions and SARS-CoV-2 IHC observed in SARS-CoV-2 (A, C, E, G, I, K, M, O) and BCG-SARS-CoV-2 (B, D, F, H, J, K, L, N, P) at 7 dpi.** These analyses were performed in specific areas like orbital cerebral cortex (precentral area) (A, B), septum (C, D), pyriform cortex (E, F), olfactory tubercle (G, H), cerebral cortex (postcentral area) (I, J), thalamus (K, L), mesencephalon (M, N) and pons (O, P). Histopathological study revealed more severe lesions in the BCG-stimulated animals, highlighting perivascular lymphocytic cuffings, increase in glial cell population (mainly microglia) and neuronal degeneration, characterized by red neurons and cytoplasmic ballooning; H&E, 10×. IHC of SARS-CoV-2 (insets) revealed a higher number of infected neurons in non-stimulated group at 7 dpi; IHC, 20×.

**Additional file 5**. **Main microscopic changes observed in the lungs of BCG-SARS-CoV-2 mice at 5–8 days post SARS-COV-2 infection.** (A) Perivascular and peribronchiolar mononuclear cell infiltration, hyperplasia of the bronchiolar epithelium and foci of pleuritis; H&E, 10×. (B) Vascular thrombosis; H&E, 20×. (C) Abundant foamy macrophages in the alveolar interstitium; H&E, 40×. (D) Desquamative alveolitis; H&E, 40×.

**Additional file 6**. **IHC evaluation (CD3, PAX5, Iba-1) in brain (A, B, C) and lungs (D, E, F) of BCG-SARS-CoV-2 mice at 5–8 days post-infection.** (A) CD3^+^ T cells immunoexpression in lymphocytic perivascular cuffings (arrowhead); anti-CD3, 40×. (B) No presence of PAX5^+^ B cells immunoexpression in lymphocytic perivascular cuffings; anti-PAX5, 40×. (arrowhead). (C) Iba-1^+^ microglial cells immunoexpression surrounding lymphocytic perivascular cuffings (arrowhead); anti-Iba-1, 20×. (D) CD3^+^ T cells immunoexpression in lymphocytic perivascular cuffings and alveolar interstitium (arrowhead); anti-CD3, 40×. (E) Minimal presence of PAX5^+^ B cells immunoexpression in lymphocytic perivascular cuffings; anti-PAX5, 40×. (arrowhead). (F) Iba-1^+^ macrophages immunoexpression in pulmonar interstitium (arrowhead); anti-Iba-1, 20×. Inset: Iba-1^+^ foamy macrophages immunoexpression in alveolar interstitium; anti-Iba-1, 40×.

## Data Availability

All data generated or analysed during this study are included in this published article [and its supplementary information files].
