# Supplementary material for: Neuropathological lesions in intravenous BCG-stimulated K18-hACE2 mice challenged with SARS-CoV-2
Source: Vet Res. 2024 May 31;55:71. doi: 10.1186/s13567-024-01325-7 (PMC11143641; doi:10.1186/s13567-024-01325-7)
Supplement: Supplementary file 1 — Additional file 1. Representation of clinical scores of infected animals at the day of sacrifice and viral loads (brain, lung, trachea/nasal turbinates) from both groups. *The clinical score from this animal could not be recorded because it was found dead at 7 dpi. **These scores correspond to the day on which the animals were euthanized which coincided with the day of the highest clinical score. [file 13567_2024_1325_MOESM1_ESM.docx]

|  | **Group 1 (SARS-CoV-2)** | | | | | | | | | | | | | | | | |  |  |  |  |  |
| --- | --- | --- | --- | --- | --- | --- | --- | --- | --- | --- | --- | --- | --- | --- | --- | --- | --- | --- | --- | --- | --- | --- |
| ID Mouse | 1.13 | 1.14 | 1.15 | 1.16 | | 1.17 | | 1.18 | | 1.19 | | 1.20 | | 1.21 | | 1.22 | |  |  |  |  |  |
| Day of sacrifice/death | 5 dpi | 6 dpi | 7 dpi | 7 dpi* | | 8 dpi | | 8 dpi | | 8 dpi | | 8 dpi | | 8 dpi | | 8 dpi | |  |  |  |  |  |
| Brain viral loads (copies/µL) | 2.61E+05 | 7.79E+04 | 7.16E+04 | 3.08E+06 | | 0.00 | | 0.00 | | 0.00 | | 0.00 | | 0.00 | | 0.00 | |  |  |  |  |  |
| Lung viral loads (copies/µL) | 3.19E+02 | 3.24E+03 | 0.00 | 0.00 | | 0.00 | | 0.00 | | 0.00 | | 0.00 | | 0.00 | | 0.00 | |  |  |  |  |  |
| Trachea/turbinates viral loads (copies/µL) | 2.55E+01 | 6.34E+00 | 5.44E+01 | 0.00 | | 0.00 | | 0.00 | | 3.65E+01 | | 6.67E+00 | | 0.00 | | 0.00 | |  |  |  |  |  |
| Loss of weight | 1 | 2 | 2 | - | | 0 | | 0 | | 0 | | 0 | | 0 | | 0 | |  |  |  |  |  |
| Hair appearance | 1 | 1 | 0 | - | | 0 | | 0 | | 0 | | 0 | | 0 | | 0 | |  |  |  |  |  |
| Level of activity | 1 | 1 | 2 | - | | 0 | | 0 | | 0 | | 0 | | 0 | | 0 | |  |  |  |  |  |
| Eye closing | 0 | 0 | 1 | - | | 0 | | 0 | | 0 | | 0 | | 0 | | 0 | |  |  |  |  |  |
| Respiratory rate | 1 | 1 | 0 | - | | 0 | | 0 | | 0 | | 0 | | 0 | | 0 | |  |  |  |  |  |
| Neurological signs | 1 | 1 | 0 | - | | 0 | | 0 | | 0 | | 0 | | 0 | | 0 | |  |  |  |  |  |
| **Total clinical score**** | 5 | 6 | 5 | - | | 0 | | 0 | | 0 | | 0 | | 0 | | 0 | |  |  |  |  |  |
|  | **Group 2 (BCG-SARS-CoV-2)** | | | | | | | | | | | | | | | | |  |  |  |  |  |
| ID Mouse | 2.12 | 2.13 | 2.14 | 2.15 | | 2.16 | | 2.17 | | 2.18 | | 2.19 | | 2.20 | | 2.21 | |  |  |  |  |  |
| Day of sacrifice/death | 5 dpi | 6 dpi | 6 dpi | 7 dpi | | 7 dpi | | 7 dpi | | 8 dpi | | 8 dpi | | 8 dpi | | 8 dpi | |  |  |  |  |  |
| Brain viral loads (copies/µL) | 1.05E+02 | 4.13E+06 | 1.20E+05 | 1.29E+04 | | 5.17E+05 | | 1.65E+05 | | 0.00 | | 0.00 | | 0.00 | | 0.00 | |  |  |  |  |  |
| Lung viral loads (copies/µL) | 1.01E+01 | 1.12E+02 | 8.24E+02 | 0.00 | | 0.00 | | 1.20E+02 | | 0.00 | | 0.00 | | 0.00 | | 0.00 | |  |  |  |  |  |
| Trachea/turbinates viral loads (copies/µL) | 6.26E+00 | 4.70E+02 | 2.05E+00 | 2.88E+01 | | 5.76E+00 | | 2.60E+02 | | 1.08E+01 | | 3.53E+01 | | 4.29E+01 | | 0.00 | |  |  |  |  |  |
| Loss of weight | 2 | 2 | 2 | 2 | | 2 | | 1 | | 0 | | 0 | | 0 | | 0 | |  |  |  |  |  |
| Hair appearance | 0 | 0 | 0 | 0 | | 0 | | 0 | | 0 | | 0 | | 0 | | 0 | |  |  |  |  |  |
| Level of activity | 2 | 1 | 1 | 2 | | 2 | | 2 | | 0 | | 0 | | 0 | | 0 | |  |  |  |  |  |
| Eye closing | 0 | 0 | 0 | 0 | | 2 | | 2 | | 0 | | 0 | | 0 | | 0 | |  |  |  |  |  |
| Respiratory rate | 1 | 0 | 1 | 0 | | 0 | | 0 | | 0 | | 0 | | 0 | | 0 | |  |  |  |  |  |
| Neurological signs | 1 | 1 | 0 | 0 | | 0 | | 0 | | 0 | | 0 | | 0 | | 0 | |  |  |  |  |  |
| **Total clinical score** | 6 | 4 | 4 | 4 | | 6 | | 5 | | 0 | | 0 | | 0 | | 0 | |  |  |  |  |  |
|  | **Group 3 (Negative control)** | | | |  | |  | |  | |  | |  | |  | |  |  |  |  |  |  |
| ID Mouse | 3.1 | 3.2 | 3.3 | 3.4 | |  | |  | |  | |  | |  | |  | |  |  |  |  |  |
| Day of sacrifice/death | - | - | - | - | |  | |  | |  | |  | |  | |  | |  |  |  |  |  |
| Brain viral loads (copies/µL) | 0.00 | 0.00 | 0.00 | 0.00 | |  | |  | |  | |  | |  | |  | |  |  |  |  |  |
| Lung viral loads (copies/µL) | 0.00 | 0.00 | 0.00 | 0.00 | |  | |  | |  | |  | |  | |  | |  |  |  |  |  |
| Trachea/turbinates viral loads (copies/µL) | 0.00 | 0.00 | 0.00 | 0.00 | |  | |  | |  | |  | |  | |  | |  |  |  |  |  |
| Loss of weight | 0 | 0 | 0 | 0 | |  | |  | |  | |  | |  | |  | |  |  |  |  |  |
| Hair appearance | 0 | 0 | 0 | 0 | |  | |  | |  | |  | |  | |  | |  |  |  |  |  |
| Level of activity | 0 | 0 | 0 | 0 | |  | |  | |  | |  | |  | |  | |  |  |  |  |  |
| Eye closing | 0 | 0 | 0 | 0 | |  | |  | |  | |  | |  | |  | |  |  |  |  |  |
| Respiratory rate | | 0 | 0 | 0 | 0 | |  | |  | |  | |  | |  | |  | |  |  |  |  |
| Neurological signs | | 0 | 0 | 0 | 0 | |  | |  | |  | |  | |  | |  | |  |  |  |  |
| **Total clinical score** | | 0 | 0 | 0 | 0 | |  | |  | |  | |  | |  | |  | |  |  |  |  |
