# Supplementary figures and images for: Neuropathological lesions in intravenous BCG-stimulated K18-hACE2 mice challenged with SARS-CoV-2
Source: Vet Res. 2024 May 31;55:71. doi: 10.1186/s13567-024-01325-7 (PMC11143641; doi:10.1186/s13567-024-01325-7)

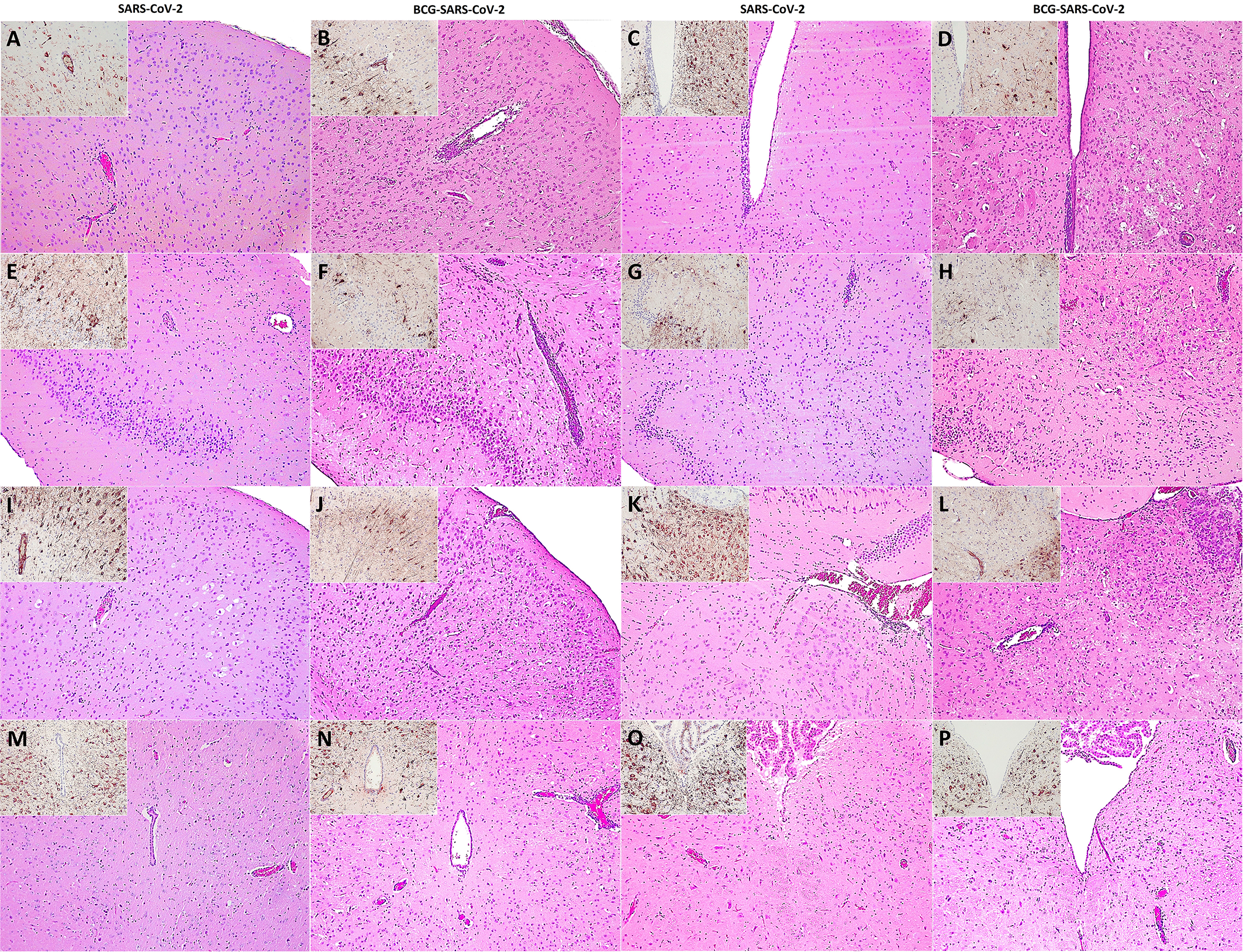

Supplement: Supplementary file 4 — Additional file 4. Brain lesions and SARS-CoV-2 IHC observed in SARS-CoV-2 (A, C, E, G, I, K, M, O) and BCG-SARS-CoV-2 (B, D, F, H, J, K, L, N, P) at 7 dpi. These analyses were performed in specific areas like orbital cerebral cortex (precentral area) (A, B), septum (C, D), pyriform cortex (E, F), olfactory tubercle (G, H), cerebral cortex (postcentral area) (I, J), thalamus (K, L), mesencephalon (M, N) and pons (O, P). Histopathological study revealed more severe lesions in the BCG-stimulated animals, highlighting perivascular lymphocytic cuffings, increase in glial cell population (mainly microglia) and neuronal degeneration, characterized by red neurons and cytoplasmic ballooning; H&E, 10×. IHC of SARS-CoV-2 (insets) revealed a higher number of infected neurons in non-stimulated group at 7 dpi; IHC, 20×. [file 13567_2024_1325_MOESM4_ESM.tif]

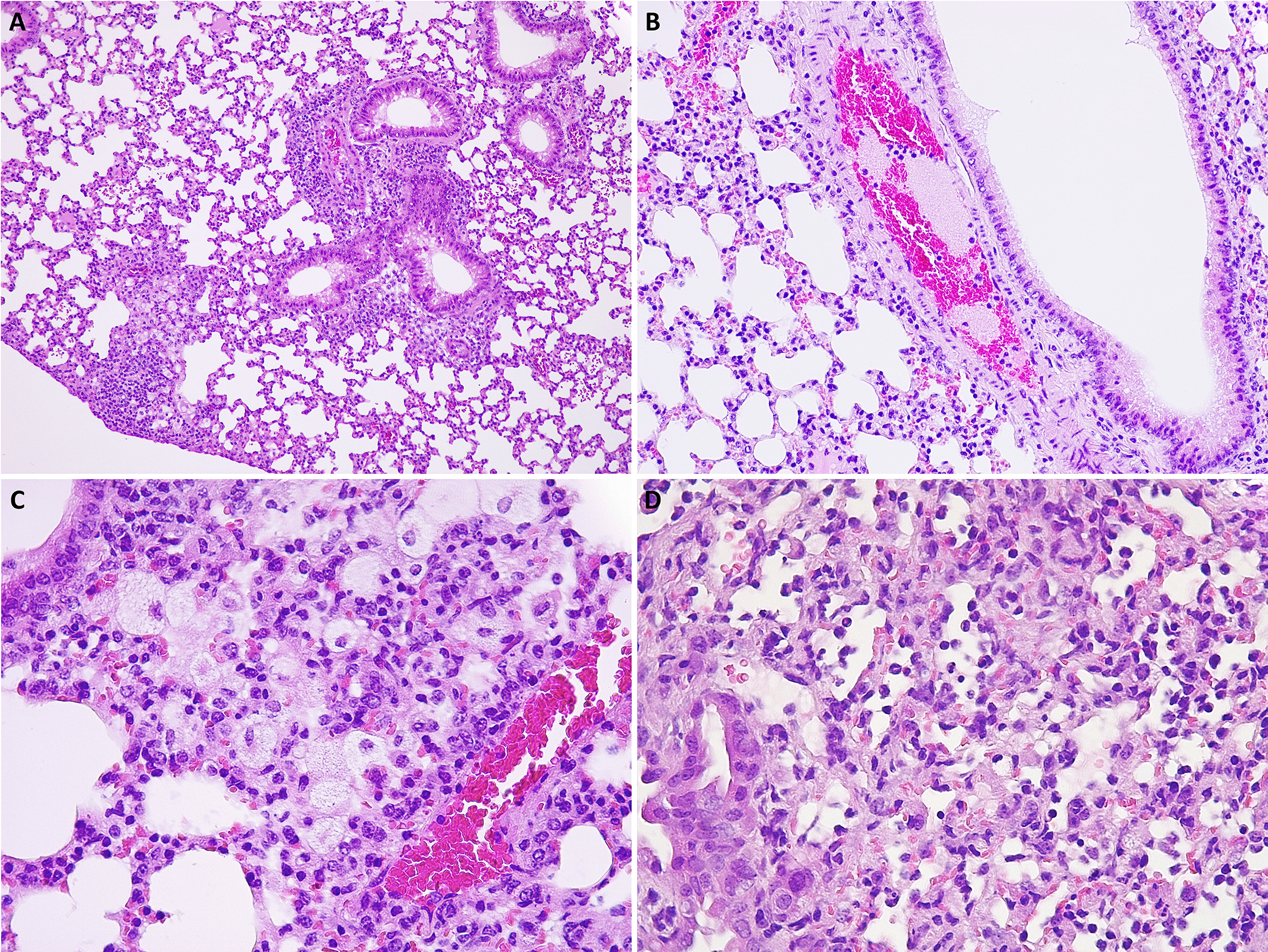

Supplement: Supplementary file 5 — Additional file 5. Main microscopic changes observed in the lungs of BCG-SARS-CoV-2 mice at 5–8 days post SARS-COV-2 infection. (A) Perivascular and peribronchiolar mononuclear cell infiltration, hyperplasia of the bronchiolar epithelium and foci of pleuritis; H&E, 10×. (B) Vascular thrombosis; H&E, 20×. (C) Abundant foamy macrophages in the alveolar interstitium; H&E, 40×. (D) Desquamative alveolitis; H&E, 40×. [file 13567_2024_1325_MOESM5_ESM.tif]

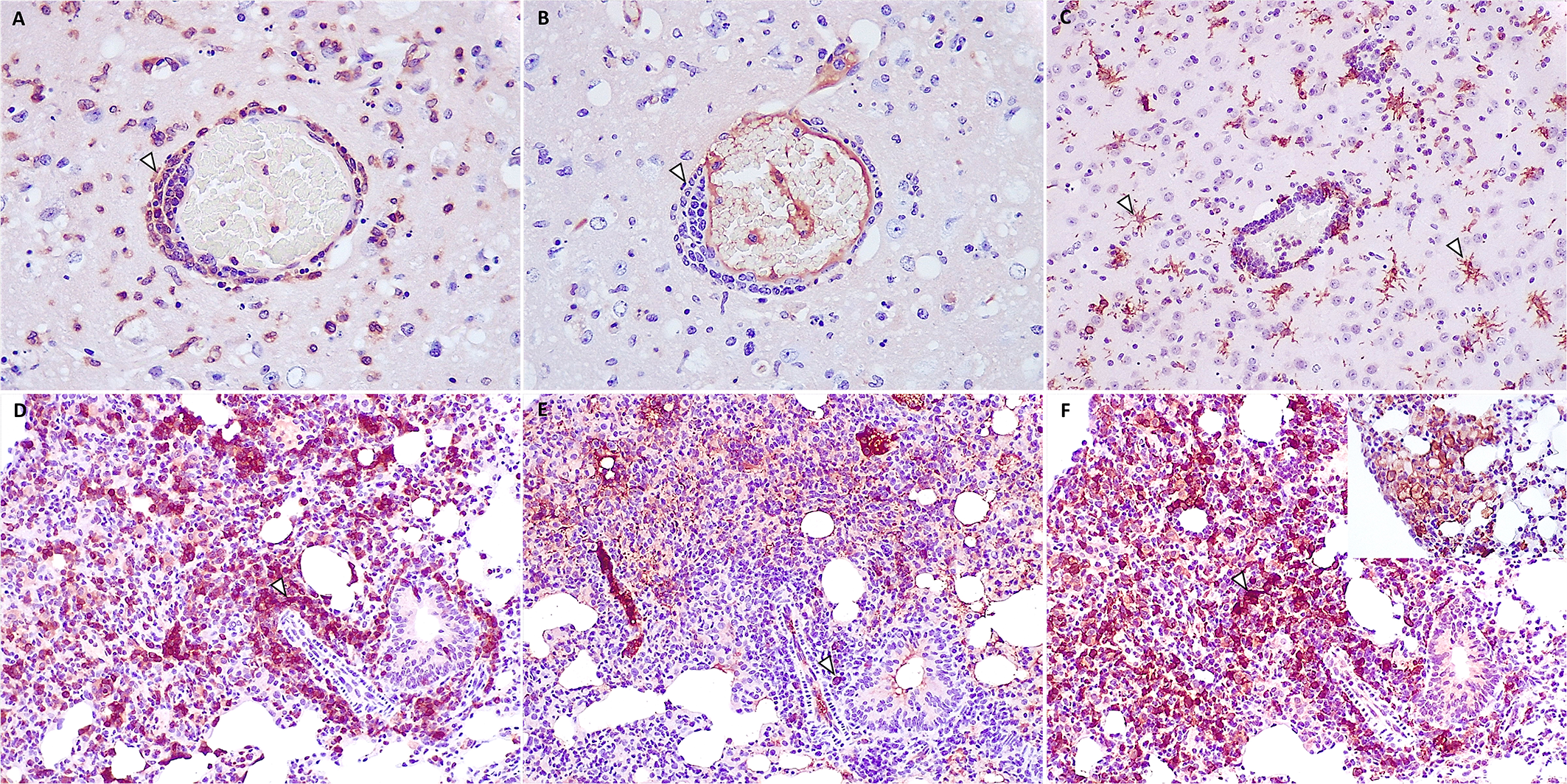

Supplement: Supplementary file 6 — Additional file 6. IHC evaluation (CD3, PAX5, Iba-1) in brain (A, B, C) and lungs (D, E, F) of BCG-SARS-CoV-2 mice at 5–8 days post-infection. (A) CD3+ T cells immunoexpression in lymphocytic perivascular cuffings (arrowhead); anti-CD3, 40×. (B) No presence of PAX5+ B cells immunoexpression in lymphocytic perivascular cuffings; anti-PAX5, 40×. (arrowhead). (C) Iba-1+ microglial cells immunoexpression surrounding lymphocytic perivascular cuffings (arrowhead); anti-Iba-1, 20×. (D) CD3+ T cells immunoexpression in lymphocytic perivascular cuffings and alveolar interstitium (arrowhead); anti-CD3, 40×. (E) Minimal presence of PAX5+ B cells immunoexpression in lymphocytic perivascular cuffings; anti-PAX5, 40×. (arrowhead). (F) Iba-1+ macrophages immunoexpression in pulmonar interstitium (arrowhead); anti-Iba-1, 20×. Inset: Iba-1+ foamy macrophages immunoexpression in alveolar interstitium; anti-Iba-1, 40×. [file 13567_2024_1325_MOESM6_ESM.tif]
